# Efficacy of selenium on patients undergoing cardiac surgery: a meta-analysis of randomized controlled trials

**DOI:** 10.1186/s13019-024-02761-4

**Published:** 2024-04-24

**Authors:** Ahmed M. Sarhan, Ahmed K. Awad, Abdullah K. Alassiri, Mohamed Sameh Abd-Alkhaleq, Rahmeh Al-Asmar, Ahmed Reda Gonnah

**Affiliations:** 1 Faculty of Medicine, October 6 University, Cairo, Egypt; 2 Faculty of Medicine, Ain-Shams University, Cairo, Egypt; 3 Faculty of Medicine, King Abdulaziz University, Jeddah, Saudi Arabia; 4 Faculty of Medicine, University of Jordan, Amman, Jordan; 5 Imperial College Healthcare NHS Trust, London, UK

**Keywords:** Selenium, Cardiac surgery, Acute kidney injury, Meta-analysis

## Abstract

**Introduction:**

Postoperative complications pose significant challenges in cardiac surgery and with the evolution of selenium as a potential anti-inflammatory agent, some studies reported its inefficiency. Thus, we conducted our meta-analysis to evaluate the impact of selenium supplementation on cardiac surgery patients.

**Methods:**

Different databases such as PubMed, Embase, and Cochrane Library from inception till January 2024 were searched identifying a total of seven randomized-controlled trials involving selenium supplementation after cardiac surgery. Risk ratio (RR) and Mean difference (MD) were calculated with a 95% confidence interval (CI).

**Results:**

The selenium intervention significantly raised the incidence of Acute Kidney injury (RR 0.76; 95% CI: 0.59, 0.98; *P* = 0.04) while significantly reducing the duration of hospital stay (MD -1.33; 95% CI: -2.51, -0.16; *P* = 0.03) and postoperative CRP levels (SMD -0.18; 95% CI: -0.34, -0.02; *P* = 0.03). The effect of selenium intervention on days spent in ICU (MD -0.01; 95% CI: -0.28, 0.25; *P* = 0.92), mortality (RR 1.07; 95% CI: 0.84, 1.37; *P* = 0.57) and incidence of hospital acquired infections (RR 0.98; 95% CI: 0.76, 1.26; *P* = 0.88) is insignificant.

**Conclusion:**

Selenium supplementation did not significantly reduce major postoperative complications in cardiac surgery patients. However, its ability to modulate inflammation, as reflected in decreased C-reactive protein levels, highlights its potential role in managing the inflammatory response. Future investigations should focus on optimized selenium supplementation strategies in conjunction with other antioxidants to enhance its benefits.

## Introduction

Cardiac surgical interventions, ranging from coronary artery bypass grafting (CABG) to valve replacements, are life-saving procedures for patients presenting with cardiovascular disease. These surgical approaches offer improved survival and quality of life, however, are associated with potential complications, affecting patients' postoperative care. Selenium (Se), an essential trace element, securing an increasing interest in the realm of cardiovascular care due to its multifaceted biological roles, including antioxidant and anti-inflammatory properties [1]. Beyond its traditional role in cellular homeostasis, selenium has emerged as a potential modulator of postoperative complications.

While prior investigations have primarily focused on selenium's ability to prevent acute kidney injury (AKI) following cardiac surgery [2], our research focuses on exploring the broader implications of selenium in mitigating a spectrum of complications that can affect cardiac surgery patients. Beyond the effect of selenium on AKI prevention, research extended the purview to critically ill cardiac surgery, major trauma, and subarachnoid hemorrhage patients. This study unveiled the potential of antioxidant supplementation, including selenium, to modulate inflammatory responses, potentially influencing a spectrum of postoperative complications. In contrast, the SUSTAIN CSX Randomized Clinical Trial challenged conventional assumptions by suggesting that high-dose selenium may not significantly influence morbidity or mortality in high-risk cardiac surgery patients [3]. Moreover, the exploration of low-dose selenium administration during coronary artery bypass graft surgery raised questions about predominant clinical effects while highlighting its safety profile [4]. Thus, we conducted our systematic review and meta-analysis to further investigate the multifaceted relationship between selenium supplementation and the reduction of complications following cardiac surgeries.

## Methods

This systematic review was performed following the PRISMA guidelines (preferred reporting items for systematic review and meta-analysis) [5]. In this review, we used EndnoteX9 [6] as a reference manager and was registered with ID: 10.17605/OSF.IO/GJXVP.

### Inclusion criteria

We included all randomized control trials that compared selenium administration (either alone or among other drugs) to control perioperatively for cardiac surgical procedures without restriction to age, sex, or language. Studies that compared selenium administration in patients undergoing less invasive cardiac procedures were excluded such as Transcatheter aortic valve implantation (TAVI) or percutaneous coronary intervention (PCI). All studies found were available in English, and the full manuscript was also available in all of the studies. We excluded papers that had minimal interventions and included only major cardiac surgeries.

### Literature search

We performed a comprehensive literature search on PubMed, Embase, Cochrane Library, Clinical trials.gov, and Scopus from inception until 20 January 2024 with keywords describing the following concepts (selenium, cardiac, and surgery): All duplicates were removed, and then all references were screened for the possibility of an eligible study that was not included.

### Screening the results

The results were screened manually by two independent authors. First, titles and abstracts were screened for eligible studies, and then the full text of papers was screened. Two independent authors performed the screening, and conflicts were resolved by discussion and consultation with a third author.

### Quality assessment

The quality assessment in this review was also performed by 2 independent authors in strict accordance with the Cochrane ROB tool [7]. This method uses 5 domains to judge each study: selection, performance, attrition, reporting, and other biases. In each domain, the study is given one of three judgments according to the risk of bias in the corresponding section (high, low, or unclear).

### Data extraction

Two groups of 2 independent authors extracted the data to a uniform data extraction sheet with crossover revision between the two groups. The data included 1) characteristics of the included studies (country, date and period of data extraction, type of study) 2) characteristics of the study groups 3) risk of bias domain 4) outcome measures. For our meta-analysis, we extracted the mortality rate, hospital stay, ICU stay, and CRP levels on different days post-operative and acute kidney injury from both groups (selenium and control).

### Heterogeneity assessment

We assessed the statistical heterogeneity using the chi-square test (Cochrane Q) and then used Cochrane Q to calculate the I^2^. Significant heterogeneity was considered when the chi-square was less than 0.1, and I^2^ higher than 50% was considered an indication of high heterogeneity. Moreover, we ran a sensitivity analysis test on all outcomes of the study in multiple scenarios in which we excluded each study one at a time and assessed the heterogeneity level to confirm that the overall effect estimate was not dependent on any single study.

### Synthesis of results

We used review manager (RevMan) [8] to conduct the meta-analysis. For the dichotomous data, we extracted the frequency of events and the total number of patients in each group and pooled them as risk ratio (RR) between the 2 groups in the inverse variance fixed-effect model. For the continuous data, we extracted the mean and standard deviation and total number of patients for each group and calculated the mean difference (MD) using the inverse variance fixed-effect model.

## Results

### Screening results

**Fig. 1:**
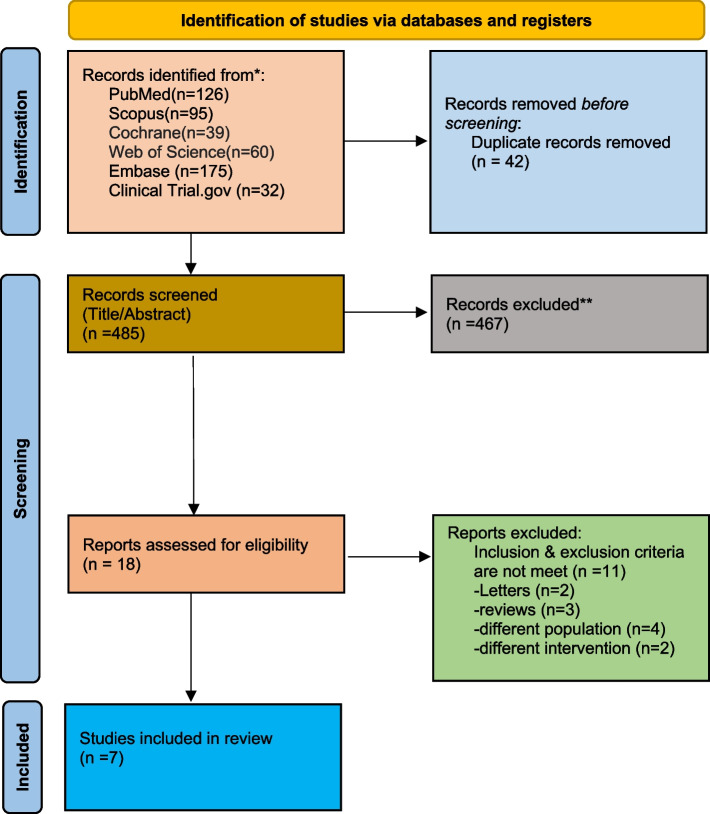
PRISMA flow chart

We identified 18 reports after removal of duplicates and multilayered screening, out of which all 7 RCTs available in full-text in the English language using selenium as an intervention in postsurgical cardiac patients against a control were included (1–4,9–11). The rest of the studies were rejected based on disagreement with the inclusion criteria. The details of the screening are shown in Fig. 1.

### Study characteristics

We reviewed and analyzed 7 RCTs comprising a total sample size of 2276 middle-aged participants, both males and females, from 7 different countries. Most of the studies were found to have adequate randomization with moderately sufficient evidence and desirable quality. Flaws were mostly observed in outcome measures, while one study had considerably compromised quality. There was no disagreement between the two authors on quality judgments. The characteristics of the included studies are shown in Table 1.

**Table 1:** Baseline characteristics and summary of included studies

study	Type study	Sample size		Age (years), Mean (SD)		Sex (male/female)		Weight (kg), Mean (SD)		Height (cm), Mean (SD)	
		Se	placebo	Se	placebo	Se	placebo	Se	placebo	Se	placebo
Shahram Amini 2018 [2]	RCT	66	71	58.21(10.54)	58.72(8.57)	NA	NA	74.93(14.01)	72.56(12.48)	166.77(9.03)	165.4(10.12)
Mette M Berger 2008 [9]	RCT	57	56	69( 8)	71(11)	70/43	70/43	75(16)	74( 17)	NA	NA
Tanja Schmidt 2018 [10]	RCT	206	205	66 (11)	68 (10)	150/56	152/53	81 (18)	80 (16)	170 (13)	170 (11)
Christian Stoppe 2023 [3]	RCT	697	697	68.0 (10.0)	68.5 (10.8)	513/184	530/167	NA	NA	NA	NA
Abbas Sedighinejad2016 [4]	RCT	41	40	54.65 (8.46)	58.2 ( 8.29)	20/21	21/19	NA	NA	NA	NA
Abbas Ali Zeraati 2021 [1]	RCT	60	60	51.1(15.2)	54.1(17.4)	NA	NA	NA	NA	NA	NA
Laaf 2022 [11]	RCT	10	10	63.8 (10.9)	61.5 (8.1)	10\0	9\1	NA	NA	NA	NA

study	Body mass index, Mean (SD)		Coronary artery disease (N)		Diabetes (N)		Ejection fraction, Mean (SD)		Doses		
	Se	placebo	Se	placebo	Se	placebo	Se	placebo	before	after	total
Shahram Amini 2018 [2]	26.92(4.62)	26.54(4.31)	6	3	26	20	53.17(7.06)	50.58(6.42)	0. 5 mg	0.5mg	1.0mg
Mette M Berger 2008 [9]	26.7 (5.3)	26.2 (4.7)	NA	NA	NA	NA	NA	NA	NA	540.4μg,and 270.2μg	1991.4 μg
Tanja Schmidt 2018 [10]	27 (5)	28 (4)	115	122	42	46	55 (17)	54 (18)	NA	4000μg	5000μg
Christian Stoppe 2023 [3]	28.9 (5.3)	28.2 (5.2)	124	108	171	173	55 (7.42)	55 (7.42)	2000 μg/L	2000 μg/L then 1000 μg/L	21,000 μg in 8 days
Abbas Sedighinejad 2016 [4]	27.16 ( 3.03)	27.32 ( 2.37)	NA	NA	NA	NA	47.31( 7.42)	47.87 ( 3.9)	600 μg	NA	NA
Abbas Ali Zeraati 2021 [1]	23.5(4.3)	25.3(3.8)	2.16	4.26	10.74	21.4	NA	NA	500 μg	500 μg	3000 μg
Laaf 2022 [11]	30.0 (3.9)	29.8 (6.9)	3	4	NA	NA	18 (5.2)	21 (6.5)	300mcg	3000mcg then 1000mcg	18000mcg

*Se* Selenium, *NA* Not available, *RCT* Randomized controlled trial

### Risk of bias analysis

**Fig. 2:**
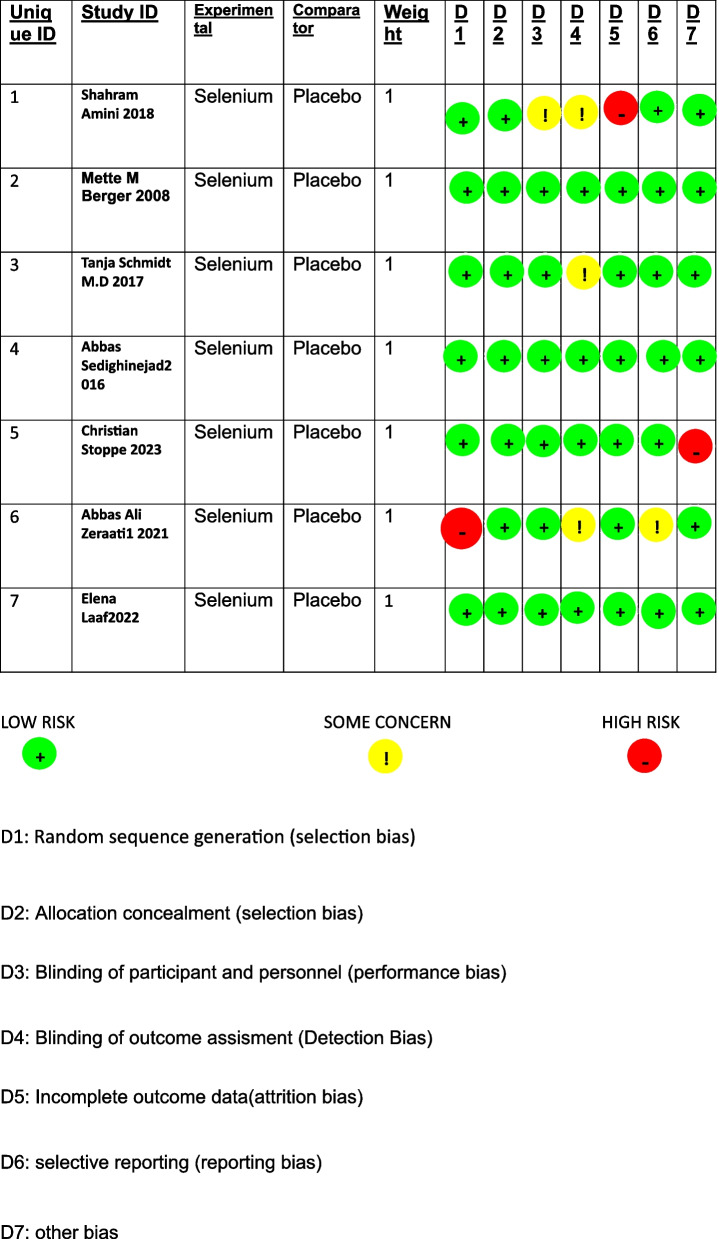
Risk of bias analysis (ROB-2)

All the studies were analyzed to be at low risk of bias except for a single study, which has some concerns for bias in multiple domains, including randomization and outcome measures, but the bias was judged to be mostly due to lack of data. The ROB analysis is shown in Fig. 2.

### Outcome measures

#### Primary Outcomes

##### Acute Kidney Injury

**Fig. 3:**
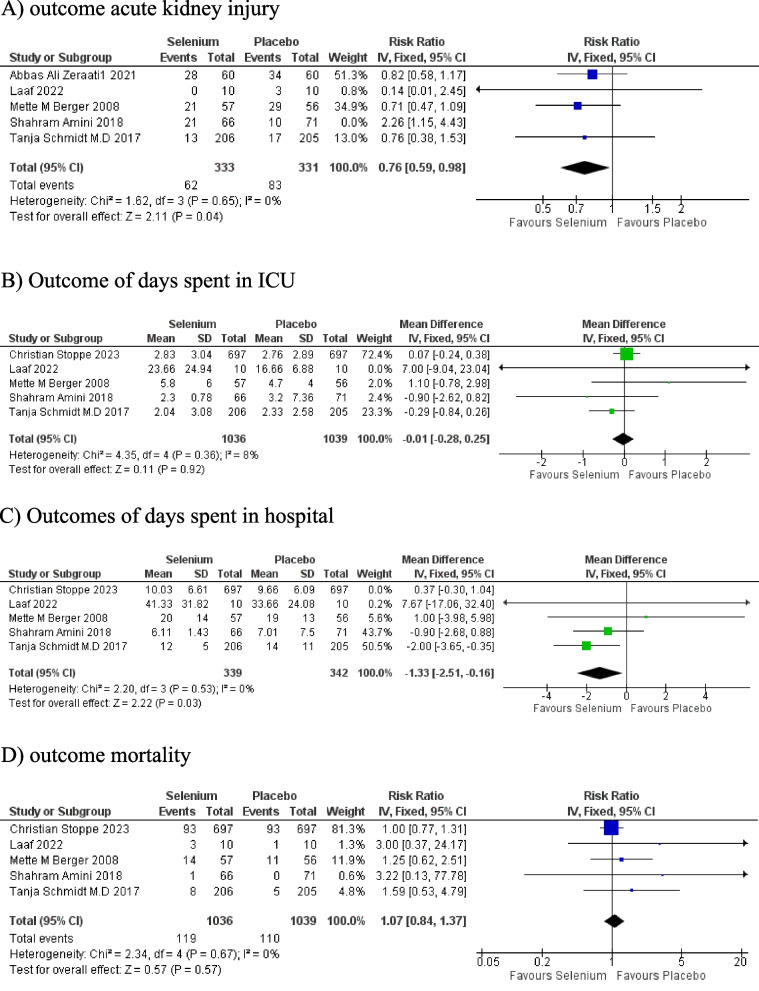
**A** Forest plot of outcome: Acute kidney injury. **B** Forest plot of outcome: Days spent in the ICU. **C** Forest plot ofoutcome: Days spent in the hospital. **D** Forest plot of outcome: mortality

The meta-analysis revealed a significant incidence of acute kidney injury in cardiac patients using selenium compared to controls (RR 0.76; 95% CI: 0.59, 0.98; *P* = 0.04). No statistical heterogeneity was detected among the studies (I^2^ = 0%) Fig. 3A.

##### Days Spent in ICU

The statistics demonstrated an insignificant association between the number of days spent in the ICU with postsurgical selenium intake (MD -0.01; 95% CI: -0.28, 0.25; *P* = 0.92). Heterogeneity was detected among the studies but not to the extent of significance (I^2^ = 8%). This heterogeneity could have arisen from minimal differences in outcome measures, sample size, and characteristics Fig. 3B.

##### Days Spent in Hospital

As per the analysis, the duration of postoperative stay in the hospital of cardiac patients using selenium was significantly different from the number of days spent in the hospital by control group patients (MD -1.33; 95% CI: -2.51, -0.16; *P* = 0.03). The analysis did not report any interstudy heterogeneity (I^2^ = 0) Fig. 3C.

##### Mortality

The statistics of postoperative cardiac patients demonstrated an insignificant association of mortality with postoperative selenium intervention (RR 1.07; 95% CI: 0.84, 1.37; *P* = 0.57). No interstudy heterogeneity was detected (I^2^ = 0) Fig. 3D.

#### Secondary outcomes

##### C-reactive Protein

**Fig. 4:**
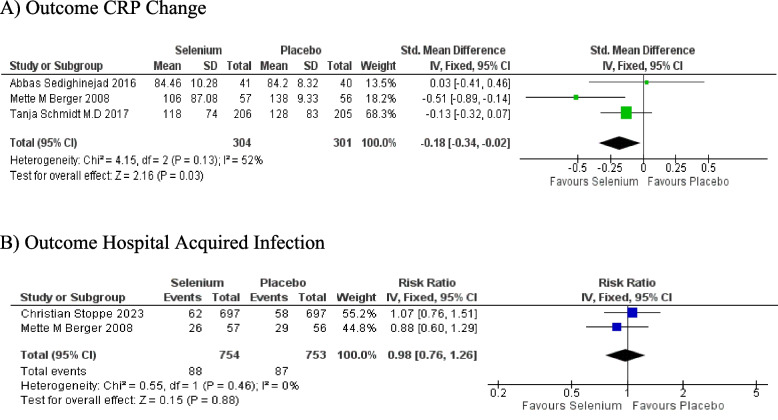
**A** Forest plot of outcome: CRP Day 0. **B** Forest plot ofoutcome:CRP Day 2. **C** Forest plot of outcome: CRP Day 3. **D** Forest plot of outcome: hospital-acquired infections

There was a significant reduction in postoperative CRP levels in the selenium intervention group compared to the control group (SMD -0.18; 95% CI: -0.34, -0.02; *P* = 0.03) after cardiac surgery. There was, however, significant heterogeneity among the studies (I^2^ = 52%) Fig. 4A-C.

##### Hospital-acquired Infections

An insignificant difference was reported between the intervention and control groups regarding the postsurgical incidence of hospital-acquired infections in cardiac patients (RR 0.98; 95% CI: 0.76, 1.26; *P* = 0.88), while no interstudy heterogeneity was detected by analysis (I^2^ = 0%) Fig. 4D.

## Discussion

Our study included 7 RCTs of moderate to high quality mostly with some concern of detection bias. Our results reported a significant incidence of acute kidney injury in patients with selenium intervention. Selenium intervention also significantly reduced the duration of postoperative hospital stay. This meta-analysis, however, did not find any significant effect on other primary outcomes by postoperative selenium intervention in cardiac patients. The strength of evidence for primary outcomes is optimal because of minimal heterogeneity and minimal bias. However, it is evident that selenium significantly lowers CRP levels and thus the inflammatory response post-surgery but interstudy heterogeneity reduced the reliability of results. The intervention did not significantly affect the incidence of hospital-acquired infections. These results are supported by highly homogenous data of significant quality and minimal risk of bias.

A study by Kamali (2019) measured hs-CRP (mg/L) four weeks postoperative and found that selenium administration for four weeks in patients undergoing CABG surgery significantly decreased serum hs-CRP levels [12].

The rationale for postoperative selenium supplementation is that cardiac surgery is followed by inflammatory activation and antioxidant depletion, causing ROS and radical accumulation and relevant adverse effects that result in multiorgan damage and a higher mortality rate as stated by previous meta-analyses [13, 14]. It has been established that selenium, a component of several antioxidant enzymes, is essential during oxidative stress due to its antioxidant and anti-inflammatory properties [14]. Diminished selenium levels have been independently associated with the development and aggravation of organ dysfunction, systemic inflammatory response, ischemic reperfusion injury, and higher mortality after cardiac surgery [15, 16]. Studies have shown that selenium is not only protective against perioperative oxidative tissue damage but can also suppress IRI-induced leukocytosis [17]. However, we have to keep in mind the nature of the cardiac procedure itself whether on or off-pump due to the high distinction in the level of stress the patients' bodies are susceptible to during on-pump compared to off-pump procedures.

Our results are therefore in agreement with the literature on the significant anti-inflammatory role of selenium after cardiac surgery [12], but our findings on mortality are contrary to some of the studies [15]. This controversy highlights the need for further evidence in this regard. A recent meta-analysis by Rehan et al. [13]  found no benefit of selenium administration post cardiac surgery, including post-operative cardiac inflammation which is an essential marker that determines post-operative patients' outcomes and recovery. The absence of benefit in other hard outcomes and mortality so far needs further investigation over a longer follow-up period and larger patient groups in future trials. Additionally, their analysis included studies that studied the efficacy of selenium as an added compound to cardioplegia which we excluded in an attempt to assess the efficacy of selenium solely.

Recent evidence suggests that selenium in combination with other metabolic and antioxidant supplements, when administered preoperatively, is far more efficacious not only in attenuating redox stress and inflammatory response but also in significantly shortening the postoperative hospital stay in both human and murine models [9, 18]. This superiority of preoperative selenium intake in combination with bioactive agents can be beneficial in the prophylaxis as well as treatment of postsurgical SIRS and in the amelioration of cardiac injury in cardiac patients, which is demonstrated by our findings [17, 19]. These speculations, however, are not supported by evidence and require further evidence-based research support. Moreover, Schmidt et al. 2017, reported an increase in some pro-inflammatory substances such as procalcitonin and bilirubin which may further raise questions marks about whether selenium has anti-inflammatory benefits [10].

There is controversy regarding the adequate dosage, time (whether preoperative or postoperative), and supplementation strategy (alone or in combination or as seleno-compound, whether bolus or continuous) of selenium in the literature as well as clinical practice [20]. Different RCTs and meta-analyses employed different strategies but reported the same outcomes [9, 12]. This accounts for the independence of the efficacy of selenium from its supplementation dosage, time, and strategy.

### Strengths and limitations

Our study is the largest up-to-date study to investigate the potential effects of selenium administered peri-operatively on patients undergoing different cardiac surgeries with the inclusion of only randomized controlled trials; However, our paper has some limitations. First, we measured inflammatory response and the effect of selenium on it by assessing the CRP values before and after administration which was reported in most of our studies except our largest included study which is Stoppe 2023. Moreover, the lack of further information about individual patients' data makes it harder to assess some clinically important outcomes such as long-term mortality. High heterogeneity was found in some of our analyses, as we propose it may be due to differences in baseline characteristics between included patients or differences in reporting cut-off values example CRP value which was reported in Schmidt 2017 day 1–5 without specification. Furthermore, the dosage of selenium wasn't consistent through our included studies and the anti-inflammatory role of selenium should need further parameters to draw a comprehensive conclusion as the studies included don't have any other inflammatory parameters to assess rather than CRP.

## Conclusion

Selenium supplementation did not significantly reduce major postoperative complications in cardiac surgery patients. However, its ability to modulate inflammation, as reflected in decreased C-reactive protein levels, highlights its potential role in managing the inflammatory response. Future investigations should focus on optimized selenium supplementation strategies in conjunction with other antioxidants to enhance its benefits.

## Data Availability

Not applicable.
